# Obesity and the Ageing Brain: Could Leptin Play a Role in Neurodegeneration?

**DOI:** 10.1155/2011/708154

**Published:** 2011-10-16

**Authors:** G. H. Doherty

**Affiliations:** School of Biology, University of Street Andrews, Bute Building, West burn Lane, St Andrews, Fife KY16 9TS, UK

## Abstract

Obesity and ageing are both characteristics of the human population that are on the increase across the globe. It has long been established that ageing is the major risk factor for neurodegenerative conditions such as Alzheimer's disease, and it is becoming increasingly evident that obesity is another such factor. Leptin resistance or insensitivity has been uncovered as a cause of obesity, and in addition the leptin signalling system is less potent in the elderly. Taken together, these findings reveal that this molecule may be a link between neurodegeneration and obesity or ageing. It is now known that leptin has beneficial effects on both the survival and neurophysiology of the neurons that are lost in Alzheimer's disease suggesting that it may be an important research target in the quest for strategies to prevent, halt, or cure this condition.

## 1. Introduction

It is a fact of life that as we age, changes occur to all body systems. The outward phenotype of the elderly is obvious with greying hair, wrinkling and thinning of the skin, and a change in posture and the fluidity of movement as joints and muscles stiffen. However, some of the more devastating changes associated with ageing are often hidden from the outside world, as neurological function declines caused by the death of critical neuronal populations. Neurodegenerative conditions arise when excessive neuronal loss occurs within a discrete region of the nervous system. Thus Parkinson's disease (PD) is linked to the large scale loss of neurons within the substantia nigra pars compacta, and Alzheimer's disease (AD) with degeneration in the hippocampus and cerebral cortex. Loss of neuronal function has a huge impact on the ability of an individual to interact with their environment, whether through the lack of motor control in PD, or though the decline in cognition, and as a consequence of this, a full range of social interaction, in AD. These changes affect not only the sufferers, but also their family and carers, and the whole of society, as the burden of care falls onto national healthcare and social care systems. 

The major risk factor for the development of neurodegenerative conditions is ageing. Therefore, as the human population ages, it has been forecast that for dementia, between the years 2001 and 2040, western countries will see a 100% increase in the number of individuals afflicted, with a 300% increase predicted for India and China [1]. Currently, neurodegenerative conditions remain incurable. At the time of writing, nearly 41 000 articles are cited within the pubmed database for which neurodegeneration is a tagged search term [2]. And yet from all this knowledge, no cures have arisen. The reason for this is the complexity of the task of curing a neurodegenerative condition. Firstly, the neurons that have been lost need to be replaced, thereafter these replacement cells need to make the correct connections with other neurons and, if applicable, with target fields outside the nervous system. The functionality of the neuronal network must also be restored, and finally the replacement neurons must be protected from the factors that triggered neurodegeneration in the first instance, such that they too do not undergo physiological decline and cell death. Given the multistage process needed to cure a degenerative condition within the nervous system, an easier target may be to find strategies that prevent the occurrence of neurodegeneration in the first place.

A plethora of risk factors, in addition to ageing, have been identified that increase the incidence of neurodegenerative diseases. These include a sedentary lifestyle [3], blows to the head (in the case of PD) [4], exposure to heavy metals such as aluminium [5], elevated plasma levels of homocysteine [6], and obesity [7], to name but a few. This review will focus on obesity as a risk factor for the development of neurodegenerative conditions, in particular the loss of cognitive function associated with Alzheimer's disease and other dementias, with an emphasis on the antiobesity hormone leptin.

## 2. Obesity and Dementia

Obesity is the excessive accumulation of fat within an organism that may be caused by genetic factors, environmental factors, or a complex interplay between the two. The resulting energy imbalance between calories consumed in the food, against calories expended in day to day living, results in the excess nutrition being stored as fat. The World Health Organisation estimated that in 2010 there were 300 million obese adults in the global population and 42 million overweight children [8]. To clarify these figures further, it is known that in developed countries, obesity affects 25–30% of adults [9, 10]. Furthermore, the incidence of obesity in the elderly is rising. In a study of 65–74 year olds in the United States, the rate of obesity increased from 27% in 1988 to 39% in 2000 [10]. These figures give us an indication of the immense scale of the occurrence of obesity. 

Investigations into a link between obesity and the onset of dementia have been widely reported in the literature. However, conflicting results have been presented. Thus, a systematic review of published papers was undertaken, and a definitive relationship between elevated body mass index (BMI) and the incidence of dementia emerged [11]. Furthermore, it has been demonstrated that carriers of the FTO allele have a slight increase in BMI as compared to noncarriers. Interestingly, possession of this allele is also linked to a decrease in brain volume in the healthy elderly [12], linking elevated BMI to central nervous system changes. It has also been established that there is a relationship between an elevated waist to hip ratio and the risk of developing a neurodegenerative condition [13], and that advancing age and elevated BMI are linked to a decrease in brain volume, but not cognition, in the middle aged [14]. Similarly a decrease in grey matter volume has been linked to adiposity in otherwise healthy postmenopausal women [15]. However, these studies did not reveal the underlying mechanisms that are activated by obesity, that then lead to degenerative changes in the brain. Furthermore, there is a lack of clarity in the published literature as to whether being overweight or just being obese is a risk factor for neurodegeneration and therefore further population-based studies, coupled with rigorous laboratory-based tests designed to tease out the underlying mechanisms linking obesity to dementia, need to be carried out.

Just as the causes of neurodegeneration per se are multiple, complex, and likely to be interlinked, the causes of the increased neurodegenerative risk induced by obesity are also multiple and complex. Roles for lipotoxicity [16], diabetes and insulin dysregulation or insensitivity [17, 18], and erroneous synthesis or response to a number of circulating factors have been postulated as potential mechanisms. It is likely that all these factors and more are important in the observed risk, but this review will solely focus on a potential role for the antiobesity hormone leptin.

## 3. The Antiobesity Hormone Leptin

Leptin is the product of the ob gene, first cloned in 1994 [19]. Expression has been detected in adipose tissue, placenta, the gastrointestinal system, and in a number of regions of the central nervous system [19–22]. It is well established that leptin plays a central role in the regulation of body weight, reducing appetite and thus an inverse relationship is established between plasma leptin levels and food intake [23, 24]. The leptin receptor, Ob-R is widely expressed in the body and is a member of the class I cytokine receptor family [25]. Alternate splicing of Ob-R gives rise to six isoforms (Ob-Ra, Ob-Rb, Ob-Rc, Ob-Rd, Ob-Re, Ob-Rf) all of which possess the extracellular leptin binding domain, but of which only Ob-Rb has signalling competency, due to the possession of the full intracellular domain [26–31]. 

**Figure 1:**
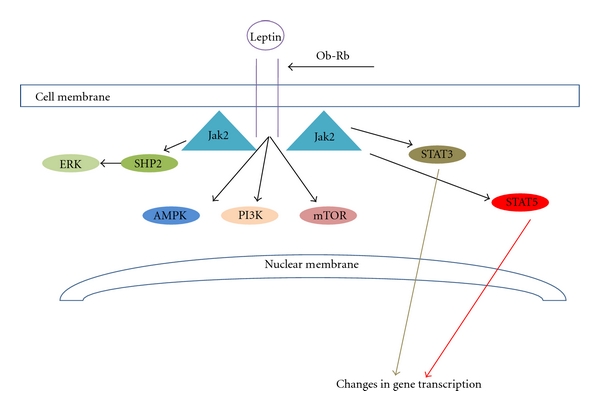
Signalling downstream of leptin binding to the Ob-R receptor. A number of pathways are activated. JAK-2-mediated phosphorylation of Tyr_985_ on Ob-Rb activates SHP2 and subsequently ERK. Phosphorylation of Tyr_1077_ and to a lesser extent Tyr_1138_ activates STAT5, whilst phosphorylation of Tyr_1138_ activates STAT3. Activation of STAT transcription factors leads to alterations in gene expression. The biochemical pathways leading to activation of PI3kinase, AMPK, and mTOR when leptin binds Ob-Rb are less well characterised.

Downstream of leptin binding to Ob-Rb, a number of intracellular signalling pathways, can be triggered (Figure 1). Ob-Rb does not possess intrinsic enzymatic activity and therefore in order to transduce the signals initiated by leptin binding, Janus kinase-2 (JAK-2) that is associated with Ob-Rb, is autophosphorylated and activated [25, 32]. Thereafter JAK-2 phosphorylates tyrosine residues within the Ob-Rb intracellular domain to mediate the downstream effects. Each tyrosine (Tyr) that is phosphorylated triggers a unique signalling event. As such, phosphorylation of Tyr_985_ leads to the recruitment of Src-homology-2 (SH2)-containing tyrosine phosphatase-2, triggering an extracellular signal-regulated kinase (ERK) signalling cascade [33]. It is of interest to note that mutagenesis of this tyrosine residue leads to adult-onset obesity linked to leptin resistance in mice [34]. Tyr_1077_ phosphorylation recruits and activates the transcription factor signal transducer and activator of transcription 5 (STAT5) [35], whilst STAT3 activation (and to a lesser extent STAT5 activation) has been linked to phosphorylation of Tyr_1138_ [33, 35]. Other signalling events known to be triggered by the binding of leptin to Ob-Rb include phosphatidylinositol 3-kinase** (**PI-3kinase), nuclear factor kappa B (NF-*κ*B) [36], and adenosine monophosphate (AMP)-dependent protein kinase [37] activity although the enzymatic pathways linking them to receptor activation remain to be fully elucidated. 

Expression of the leptin receptor has been described in the brain, with transport of leptin into the brain believed to be achieved by leptin binding to the short forms of the receptor Ob-Ra and Ob-Rc that are present in the choroid plexus and the microvasculature of the brain [26]. The signalling of leptin within the central nervous system is believed to predominantly be via the full length Ob-Rb form of the Ob receptor thereafter activating the signalling pathways described in the previous paragraph. Expression of Ob-Rb has widely been identified within the central nervous system including the cortex and hippocampus that are the major site of neurodegeneration in AD and the substantia nigra that is the major site of neuronal loss in PD [27, 30, 38]. To date there is very little known about regulation of the leptin receptor under the specific circumstances of a given neurodegenerative disorder, and this will clearly be a very important area of research in the future. It is known that both a deficiency in circulating leptin, as observed in leptin-deficient animals, and an inability to respond to leptin, as observed in animals with deficits in the Ob receptor, lead to upregulation of Ob-Rb suggesting that there is close interplay between levels of leptin detected and expression of the full length receptor [39, 40]. 

A myriad of research has been conducted demonstrating that leptin, coupled with a correct response to the hormone, is central to the control of bodyweight and to other physiological processes. Thus adipocyte-generated leptin acts as a satiety signal inhibiting food intake [39] and in addition, evidence suggests that leptin plays a key role in energy expenditure. Thus, mice that fail to respond to leptin develop obesity even if fed a diet that restricts calorific intake to normal levels [41] implying that these animals are not expending the energy from their food efficiently. Therefore, a reduced ability to synthesise or respond to leptin is likely to lead to a failure to control appetite and reduced expenditure of energy, leading to an increase in weight and, in time, obesity. In ob/ob mice, a loss of function mutation in the ob gene that encodes leptin leads to obesity by around four weeks of age [42]. Likewise db/db mice or fa/fa rats that carry a loss of function mutation in the Ob-R gene are obese by a similar age [42]. It is therefore not surprising that failures in the leptin signalling system have been linked to obesity in humans [43–45]. Mechanisms by which this failure can occur include deficits in leptin transport across the blood-brain barrier, insufficient leptin release and mutations in the Ob-R receptor, or defects in its trafficking or downstream signalling, preventing the correct response to the circulating hormone [7].

## 4. Leptin and Neurodegeneration

Neurodegenerative diseases, as mentioned above, are characterised by the loss of neurons in a specific region of the nervous system and with abnormalities of neuronal function in the cells that remain. It has been widely reported that we face an increasing demographic shift across the globe to an ageing population [46], which will bring about an increase in the incidence of age-related neurodegeneration. Given that we are also facing increasing levels of obesity in the elderly [11], if there is a link between obesity and neurodegeneration, then we need to know what it is and how to deal with it. There is now a range of research data published that reveals that leptin is one molecule that is involved in linking obesity to neurodegeneration.

It has been demonstrated that aged rats have a lower response to leptin as compared to young rats with decreased signalling downstream of the Ob-R receptor identified as a reason for this, and in particular a decrease in STAT3 activation has been noted with age [47]. In addition to the age-linked reduced ability to respond to leptin, there is also an age-linked decrease in the uptake of leptin by hypothalamic nuclei, which correlates to a decrease in Ob-R expression [48]. Another mechanism that influences the age-related decline in leptin responsiveness is an increase in the levels of the suppressor of cytokine signalling-3 (SOCS-3) that inhibits the transduction of the signals triggered by leptin binding to Ob-R [49]. Thus, increased SOCS-3 expression has been identified in the aged hypothalamus [50]. Similarly, enhanced hypothalamic levels of protein tyrosine phosphatase 1B are believed to contribute to age-related leptin resistance [51]. Thus, the elderly are less able to respond to leptin for a variety of physiological reasons, which raises the question of what this decrease in leptin signalling might mean for neurons.

One of the physiological properties of leptin that is central to its potential role in neurodegeneration is the discovery that leptin can prevent neuronal death. Thus leptin can protect cultured dopaminergic neurons from the central nervous system, and trigeminal sensory neurons from the peripheral nervous system, from cell death in response to a number of different stimuli [36]. In accordance with this it has been noted that the brains of leptin-deficient mice are of lower weight than their wild-type counterparts, perhaps revealing that a lower number of neurons survive in these animals [52]. In cultured murine cortical preparations, leptin can prevent excitotoxic neuronal loss triggered by NMDA. Furthermore, systemically delivered leptin can prevent excitotoxic damage *in vivo*, reducing cortical lesions in mice given high doses of ibotenate [53]. Neuroprotective roles for leptin have also been found in animal models of stroke where leptin administration protects against both oxygen-glucose deprivation and middle cerebral artery occlusion [54]. Similarly leptin is neuroprotective in both *in vivo* and *in vitro* models of PD [36, 55], and it protects hippocampal neurons in models related to epilepsy [56]. In the case of AD, transgenic mice that exhibit neurodegeneration in brain areas linked to AD have a marked reduction in the number of dying neurons following treatment with a lentiviral vector encoding leptin [57]. Taken together there is a large body of evidence to suggest that leptin may be beneficial in the prevention of neuronal death in neurodegenerative situations. 

In addition to neuronal death, conditions such as AD are characterised by alterations in neuronal function. Thus, leptin can modulate synaptic plasticity via enhancing N-Methyl-D-aspartate (NMDA) receptor function [58, 59]. Given that synaptic plasticity is studied as the cellular basis of memory formation, these findings raise the possibility that leptin can have beneficial effects on the memory loss that is perhaps the best known symptom of AD. Thus rodents that have mutations in Ob-R show memory impairment [60]. Furthermore, transgenic mice that exhibit deficits in memory and are used to model AD show a reduction in AD-linked pathological changes and an enhancement in memory if treated with exogenous leptin [61]. Whilst the discovery that leptin can influence both memory and neuronal viability in a laboratory setting strongly suggests that it may be important in either the pathogenesis of or the treatment of AD, the case for further study is only truly strengthened with human data.

Thus studies of the individuals that form the Framingham cohort have revealed that decreased circulating leptin levels are predictive of an increased risk of developing dementia or AD [62]. In contrast to this, cross-sectional studies have not revealed a link between leptin levels and vascular dementia [63], and carriers of an Ob-R mutation that reduces leptin binding do not have an enhanced risk of AD [64]. Another study looked at vascular dementia and AD in isolation. Once again this demonstrated a link between low leptin levels and the development of AD when compared to nondemented or vascular dementia patients. These latter two groups had similar leptin levels. This study exhibits that AD and vascular dementia [65], which are often grouped together as disorders, may have a very different pathogenesis with regards to the action of this antiobesity hormone. 

It is becoming increasingly evident that neuroinflammation is an important part of the pathology of neurodegenerative conditions [66]. Thus, it is crucial to determine whether leptin influences the activity or activation of the astrocytes and microglia of the central nervous system. It has been determined that glial cells may play a central role in synaptic inputs into the hypothalamus and that leptin can influence this process. It is particularly interesting to note that chronic leptin administration leads to astrocyte activation suggesting that the potential role of these cells in modulating obesity and neurodegeneration needs to be more extensively investigated in the coming years [67].

## 5. Discussion

Articles in the press often highlight so-called “epidemics” that it is believed will greatly impact upon the way that healthcare and social care systems are organised as we move forward in time. One of these issues that is frequently mentioned is ageing, another is obesity. It is becoming increasingly apparent in the scientific literature that a common consequence of both of these so-called “epidemics” is neurodegeneration. Thus, it is worthwhile to explore whether there are shared mechanisms that link these two risk factors to neurodegeneration with a view to preventing or treating illnesses such as AD. Within this framework leptin appears as one of the candidate molecules that might link obesity and age to neurodegeneration.

It is clear that obesity increases with age and that one of the reasons for this is that the way in which the body responds to leptin changes with age. Thus there is a decrease in circulating leptin [68], Ob-R, and in leptin uptake [48], coupled with an increase in inhibitors of leptin signalling [49–51]. It is well established that leptin is a satiety signal and thus a decrease in the response to it will decrease satiety, increasing energy intake and thus risking obesity. It is also well documented that leptin has many beneficial effects on both neuronal survival and neurophysiology, thus this decrease in leptin signalling in the elderly will have a negative impact on the function of neuronal networks that are sensitive to its effects. Given that the hippocampus has been long established to be fundamental to memory processes that are lost in AD, the scientific evidence that leptin is important for both the viability [56] and functioning [58, 59] of hippocampal neurons strongly suggests that leptin may be important in the pathogenesis of AD. Coupled to this is the finding from the Framingham cohort that low levels of circulating leptin are a risk factor for the future development of AD [62]. 

Since it is clear that leptin is a strong candidate as one of the molecules that links obesity to neurodegeneration, and we know that obesity in midlife enhances the risk of AD [69], the possibility of manipulating leptin levels or leptin signalling in advance of the onset of AD symptoms arises. Thus as a preventative measure, adults with midlife obesity could be assessed for their levels of, and response to, leptin and strategies put in place to boost this signalling system. In addition, restoring leptin function could have beneficial effects outwith the central nervous system including energy homeostasis and neuroendocrine function [70]. However, leptin is just one factor relating to obesity that may be involved in AD pathogenesis. In addition to leptin, other obesity-related factors are linked to AD including insulin [18], changes to the cerebral vasculature [71], and direct lipotoxicity [17]. In support of the notion that leptin may not be a cure-all for all cases of dementia is the finding that there is no correlation between leptin levels and vascular dementia [63, 65]. Nonetheless, there is strong evidence that leptin signalling has beneficial consequences for neural networks, and therefore introducing corrective strategies in individuals in whom this is compromised can only be of benefit. Furthermore, given that leptin is only one of the links between obesity and AD, any targeting of leptin signalling in obese individuals who have leptin deficiency or reduced sensitivity is likely to be most successful when combined with lifestyle changes targeting a healthy weight and level of activity. Of course it should also be emphasised that individuals who exhibit a high level of leptin insensitivity may be completely insensitive to leptin or leptin-derived therapies if treatment is not carried out alongside therapies to reverse this state of insensitivity. 

There are good arguments to be put to promote leptin and its downstream signalling pathways as possible drug targets for not only the treatment of AD but also of other neurodegenerative disorders [7, 72]. However, the ability of leptin to protect against cell death is not universal, and it has been demonstrated that leptin exerts neuroprotective effects on cerebellar Purkinje but not granule neurons in culture [73]. It is therefore crucial to investigate leptin's potential therapeutic effects extensively against each condition of interest. As such a word of caution must be raised against the current findings that imply that leptin is neuroprotective in models of AD. There are numerous mice models for AD, none of which provide a perfect recreation of the disease pathology observed in human AD patients. For example, although the Tg2576 transgenic mouse models do show the glial-mediated inflammatory response and elevated levels of A*β* and associated plaques that occur in the brains of AD sufferers [74], there are other pathologies associated with AD, such as neuronal loss and neurofibrillary tangles, that are not displayed in this model. Thus leptin's effects must be robustly tested in as many different AD rodent and *in vitro* models as possible before a definitive conclusion about how it might confer neuroprotection can be drawn. This would also help to elucidate where within the temporal progression of AD leptin might confer the maximal benefits.

In this regard, leptin's antiapoptotic actions would not offer any potential to cure AD. However, if given before symptoms occur, leptin-targeting medicines could prevent the loss of neurons associated with AD in the first instance. It could also be targeted to early stage sufferers in order to prevent further neuronal loss and therefore halt or slow down disease progression. Another possible benefit of leptin's antiapoptotic abilities is in protecting grafted tissue, whether from embryonic stem cells or fetal sources, once it is transplanted into the sufferer. Grafting of host tissue has been used with some success as a potential treatment for PD but one of the problems with this therapy is that a large number of the transplanted neurons degenerate within the host. Thus for just one PD patient, tissue from between 9 and 12 embryos is required for bilateral implantation [75]. For PD, interest has therefore turned to neurotrophins that can enhance dopaminergic neuron number in grafted tissue such as glial cell line-derived neurotrophic factor [76] or, for tissue from early embryos, tumour necrosis factor alpha (TNF-*α*) [77]. It is feasible therefore that leptin could offer neurotrophic support to grafted tissue in AD suffers if technology advances to a point where such transplants become a treatment option for this disease.

In addition to its antiapoptotic effects, leptin has also demonstrated beneficial effects on memory [78]. Thus leptin administration, again particularly early in disease progression, may help to alleviate the alterations in synaptic function associated with AD. There is a growing body of evidence that synaptic alterations may precede neuronal death in AD and therefore leptin's effects on these may be central to allowing individuals to halt the decline in memory function so strongly associated with AD. Thus leptin exerts its beneficial effects on the nervous system at multiple levels from neuronal survival to synaptic plasticity, suggesting that it could benefit AD sufferers in a number of ways. Specifically it has been determined that leptin inhibits hippocampal neurons, that are afflicted in AD, by activating BK channels leading to the initiation of a PI3-kinase signalling cascade [79]. Furthermore, leptin can evoke NMDA-receptor dependent-long-term depression under conditions of enhanced excitability [80]. Thus electrophysiological data suggests that leptin can enhance the cellular processes thought to underpin memory formation. 

One of the major benefits that leptin offers as a potential therapeutic target is that it is currently licensed for human use as an antiobesity drug. The possession of this license would markedly speed up the drug discovery pipeline [7]. Furthermore, we all produce leptin in our bodies as a naturally occurring substance and as such it is well tolerated in a therapeutic setting and few side effects have been identified following its use as an antiobesity agent. However, leptin is not an easy drug to administer, and the current treatment for obesity involves giving subcutaneous injections of the drug. Pharmacological leptin has so far been given to patients for peripheral metabolic disorders and to modulate behaviours and endocrine pathways in the hypothalamus. Thus, it is unclear whether subcutaneous administration of leptin could act on the hippocampus and cortex that are affected in AD. In rodents intranasal administration of leptin successfully raises brain leptin levels, particularly in the hypothalamus [81, 82]. Also, a nonreplicative, nonimmunogenic and non-pathogenic recombinant adeno-associated virus encoding the ob gene has been used as a form of central gene therapy by direct injection into the affected brain area to provide a stable supply of leptin in that region. This has been used successfully to suppress weight gain in prepubertal rats [83], to inhibit fat deposition [84], and to block high fat diet-induced weight gain [85]. Thus, much important work still needs to be carried out to reveal the most effective method of leptin delivery into regions of the brain in humans.

In conclusion, with an increasing elderly population and an increasing rate of obesity within that population, it is imperative that we fully understand the noted links between obesity and neurodegeneration. Candidate molecules for explaining this connection are being discovered all the time, and it is within this framework that interest has turned to the antiobesity hormone leptin. The multilevel beneficial effects that are reported for leptin in neurons give hope that we may be on the road to uncovering pathways that can be targeted to halt, prevent, and perhaps eventually cure neurodegenerative disorders such as AD.

## Abbreviations

AD: Alzheimer's disease
AMP: Adenosine monophosphate
BMI: Body mass index
ERK: Extracellular signal-regulated kinase
JAK-2: Janus kinase-2
NF-*κ*B: Nuclear factor kappa B
NMDA: N-Methyl-D-aspartate
PD: Parkinson's disease
PI3-kinase: Phosphatidylinositol 3-kinase
SOCS-3: Suppressor of cytokine signalling-3
STAT: Signal transducer and activator of transcription
TNF-*α:*: Tumour necrosis factor alpha
Tyr: Tyrosine.
